# Data on residual stresses of mooring chains measured by neutron diffraction and hole drilling techniques

**DOI:** 10.1016/j.dib.2020.105587

**Published:** 2020-04-20

**Authors:** Ershad P. Zarandi, Tung L. Lee, Bjørn H. Skallerud

**Affiliations:** a Department of Structural Engineering, Norwegian University of Science and Technology (NTNU), Richard Birkelands vei 1A, 7491, Trondheim, Norway; b ISIS Neutron Source, Science and Technology Facilities Council, Rutherford Appleton Laboratory, Harwell Campus, OX110QX, United Kingdom

**Keywords:** Residual stress, Offshore mooring chain, High strength steel, Neutron diffraction, Hole drilling

## Abstract

Residual stresses in large offshore mooring chains have been measured for the first time and presented in this article. Two chain links with the same size and material, one only subjected to proof load and no cyclic service loads and the other exposed to service loads as well as the proof load, were selected for the experiment. Residual stresses just below the surface were measured using the hole-drilling technique and the neutron diffraction technique was employed for deeper measurements. The data can be used to investigate residual stress redistribution in the chain links because of material removal due to corrosion and cyclic service loads that the chains are exposed to during their service time. Moreover, the data can be used to validate numerical models for predicting residual stresses. A more detailed interpretation of the data presented in this article is provided in “Experimental and numerical study of mooring chain residual stresses and implications for fatigue life” [1].

Specifications table

**Table:** 

Subject	Engineering
Specific subject area	Offshore engineering
Type of data	Table
How data were acquired	Neutron diffraction, ENGIN-X neutron diffractometer at STFC Rutherford Appleton Laboratory, United Kingdom
	Hole drilling, strain rosette type 1-RY61-1.5/120K, electronic measuring system (Spider8), inverted truncated-cone shaped with flat ends end mills (1-SINTCTT/1), MTS-3000 instrument at SINTEF, Norway
Data format	Raw and Analyzed
Parameters for data collection	Two chain links with the same size and material, one only subjected to proof load and no cyclic service loads and the other exposed to service loads as well as the proof load, were selected for the experiment.
Description of data collection	Residual stresses at several locations on two chain links were measured using the neutron diffraction and hole drilling techniques.
Data source location	Department of structural engineering
	Norwegian university of science and technology (NTNU)
	Trondheim, Norway
Data accessibility	With the article
Related research article	Ershad P. Zarandi, Bjørn H. Skallerud, Experimental and numerical study on mooring chain residual stresses and implications for fatigue life, International Journal of Fatigue, https://doi.org/10.1016/j.ijfatigue.2020.105530

## Value of data

• Residual stresses in large offshore mooring chains have been measured for the first time and can be used for the validation of numerical/analytical models for predicting residual stresses.
• Data can be used in the revision of offshore mooring chains design guidelines/standards.
• The fatigue life estimation of offshore mooring chains can be taken to an advanced level using the presented data.
• Data can be compared with the measurements made by other measurement techniques e.g. X-ray or deep hole drilling.
• Data can be used as an educational tool for learning how to compute residual stresses from the raw data obtained by the neutron diffraction and hole drilling techniques.

## 1 Data description

**Fig. 1:**
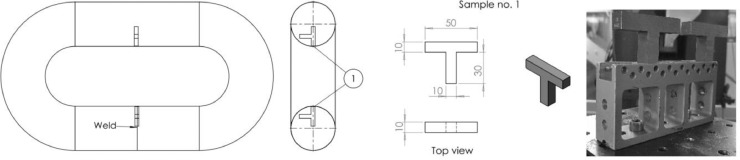
Details of the reference samples for the neutron diffraction technique.

**Fig. 2:**
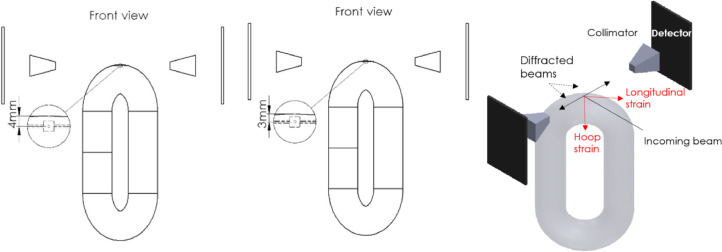
Schematic of the neutron beam paths at the chain crown for measuring hoop and longitudinal residual stresses.

**Fig. 3:**
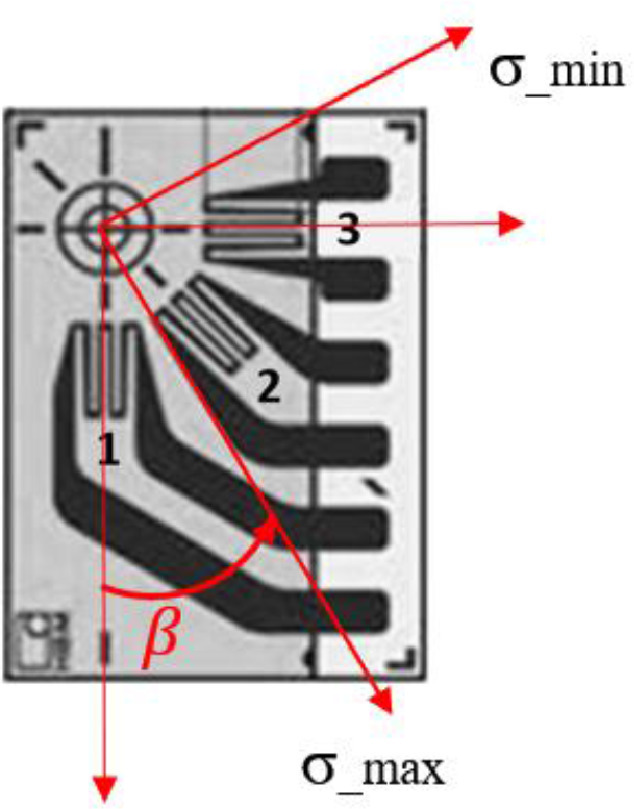
Details of the strain rosette used in the hole drilling technique.

The raw and analyzed data on the residual stresses (RS) of offshore mooring chains are presented in this article. RS at different locations on two chain links were measured using two different techniques; Neutron Diffraction (ND) and Hole Drilling (HD). Fig. 1 provides details of the reference (stress-free) samples cut from a chain link and used for the ND technique. The lattice spacings of the stress-free samples cut from the chain material are listed in Table 1. Fig. 2 illustrates a schematic of the neutron beam paths at the chain crown for measuring hoop and longitudinal RS. The raw data from the ND technique (lattice spacing *d*) and the computed strains (ɛ) using the procedure explained in Section 3.1.1 are provided in Table 2. The corresponding calculated RS (*σ*) are presented in Table 3. Fig. 3 provides the details of the strain rosette used in the HD technique. The raw data obtained by HD technique test (ɛ_1_, ɛ_2_, and ɛ_3_) on the chain links and corresponding RS along the strain gage axes (*τ*_1_, *τ*_2_, *τ*_3_, *σ*_1_, *σ*_2_, *and* *σ*_3_) as well as maximum and minimum principal stresses (*σ_max_* and *σ_min_*) and *β* angles calculated following the procedure explained in 3.1.2 are provided in Tables 4, 6, and 8 for the unused chain link and in Tables 5, 7, and 9 for the used chain link.

**Table 1:** Lattice spacing for the reference (stress-free) samples averaged on the three considered axes (radial, hoop, longitudinal).

	*d*_0_ (averaged)	Uncertainty (Δ*d*_0_ averaged)
Base	2.86783	0.000057
Weld	2.86789	0.000072

**Table 2:** Neutron diffraction data, measured d-spacings and corresponding computed residual strains.

Location	Position below the surface [mm]	Measured Lattice Parameter,*d* [Å]						Strain [µɛ]					
		Radial (R)		Hoop (θ)		Longitudinal (Z)		ɛ_*R*_		ɛ_*θ*_		ɛ_*Z*_	
		*d*	Δ*d*	*d*	Δ*d*	*d*	Δ*d*	Δɛ	Δɛ	ɛ	Δɛ	ɛ	Δɛ
used_Loc3	3			2.86664	0.000164	2.86339	0.000210			−414.9	60.5	−1548.2	75.9
used_Loc4	3	2.86883	0.000105	2.86622	0.000132	2.86253	0.000109	348.7	41.7	−561.4	50.1	−1848.1	42.9
used_Loc4	4	2.86882	0.000088	2.86638	0.000198	2.86283	0.000159	345.2	36.6	−505.6	71.8	−1743.5	58.9
used_Loc2	3			2.86640	0.000098	2.86492	0.000212			−519.5	42.4	−1035.6	78.1
used_Loc5	3	2.86959	0.000070	2.86636	0.000164	2.86578	0.000101	592.8	35.0	−533.5	62.4	−735.7	43.2
used_Loc5	4	2.86898	0.000078	2.86549	0.000233	2.86332	0.000198	380.1	37.0	−836.9	85.0	−1593.5	73.4
used_Loc1	3	2.8688	0.000071	2.86702	0.000158	2.86264	0.000133	338.2	31.8	−282.4	58.6	−1809.7	50.4
used_Loc1	4	2.86859	0.000063	2.86811	0.00021	2.86279	0.000171	265.0	29.6	97.6	75.9	−1757.4	62.8
unused_Loc3	3			2.86757	0.000141	2.86503	0.000122			−90.7	53.0	−976.3	46.9
unused_Loc4	3	2.86902	0.000081	2.86705	0.000197	2.86238	0.000312	414.9	34.5	−272.0	71.5	−1900.4	110.6
unused_Loc4	4	2.86858	0.000092	2.86757	0.000262	2.86059	0.000260	261.5	37.7	−90.7	93.5	−2524.6	92.8
unused_Loc2	3			2.86775	0.000160	2.86026	0.000214			−48.8	61.2	−2660.5	78.7
unused_loc5	3	2.86991	0.000066	2.86669	0.000049	2.86363	0.000082	704.4	34.1	−418.4	30.4	−1485.4	38.0
unused_loc5	4	2.86906	0.000088	2.86644	0.000044	2.86321	0.000069	408.0	39.7	−505.6	29.4	−1631.9	34.7
unused_loc1	3	2.86925	0.000075	2.86845	0.000154	2.86213	0.000118	495.1	32.9	216.2	57.3	−1987.6	45.7
unused_loc1	4	2.86908	0.000066	2.86805	0.000132	2.86178	0.000122	435.9	30.4	76.7	50.1	−2109.6	46.9

**Table 3:** Neutron diffraction data, computed residual stresses.

Location	Position below the surface [mm]	Stress [MPA], based on 3 axes measurements						Stress [MPA], based on 2 axes measurements (i.e. $\varepsilon_{R}=0$ in Eq. (1))					
		Radial (R)		Hoop (θ)		Longitudinal (Z)		Radial (R)		Hoop (θ)		Longitudinal (Z)	
		*σ*	Δ*σ*	*σ*	Δ*σ*	*σ*	Δ*σ*	*σ*	Δ*σ*	*σ*	Δ*σ*	*σ*	Δ*σ*
used_Loc3	3							−234.0	16.3	−199.7	18.9	−379.8	21.3
used_Loc4	3	−190.2	10.9	−334.8	11.6	−539.3	11.0	−287.2	11.1	−253.3	14.3	−457.8	13.1
used_Loc4	4	−172.1	12.9	−307.3	15.5	−504.0	14.4	−268.1	15.6	−233.5	20.3	−430.3	18.3
used_Loc2	3							−185.4	14.4	−188.5	14.9	−270.5	20.6
used_Loc5	3	13.6	11.1	−165.4	13.2	−197.6	11.6	−151.3	12.6	−171.2	17.1	−203.4	14.1
used_Loc5	4	−184.0	15.0	−377.4	18.3	−497.6	17.3	−289.7	18.9	−298.5	24.3	−418.8	22.5
used_Loc1	3	−155.3	10.9	−253.9	12.8	−496.7	12.1	−249.4	13.0	−187.4	16.7	−430.1	15.4
used_Loc1	4	−124.1	12.9	−150.7	16.1	−445.5	15.0	−197.8	16.5	−97.5	21.5	−392.3	19.4
unused_Loc3	3							−127.2	11.9	−87.1	15.2	−227.8	14.3
unused_Loc4	3	−143.5	16.9	−252.7	19.0	−511.5	22.2	−258.9	21.7	−191.2	23.8	−450.0	30.0
unused_Loc4	4	−239.0	17.1	−295.0	20.8	−681.8	20.7	−311.7	22.2	−192.5	27.5	−579.3	27.4
unused_Loc2	3							−322.9	16.7	−192.3	19.3	−607.3	22.0
unused_loc5	3	−31.0	8.5	−209.5	8.2	−379.0	8.8	−226.9	8.2	−196.2	9.5	−365.7	10.7
unused_loc5	4	−141.3	9.0	−286.5	8.3	−465.5	8.6	−254.8	7.6	−225.9	9.0	−404.9	9.9
unused_loc1	3	−73.4	10.6	−117.8	12.4	−468.0	11.4	−211.1	12.3	−86.3	16.1	−436.5	14.3
unused_loc1	4	−121.1	9.9	−178.2	11.3	−525.6	11.0	−242.3	11.6	−126.3	14.6	−473.7	14.1

**Table 4:** Hole drilling data at the crown (location 1) of the unused link.

Depth	ɛ_1_	ɛ_2_	ɛ_3_	*σ_min_*	*σ_max_*	*β*	*σ* on 1	*τ* on 1	*σ* on 2	*τ* on 2	*σ* on 3	*τ* on 3
[mm]	[*μ*ɛ]	[*μ*ɛ]	[*μ*ɛ]	[*MPa*]	[*MPa*]	[°]	[*MPa*]	[*MPa*]	[*MPa*]	[*MPa*]	[*MPa*]	[*MPa*]
0.025	2.47	0.25	−0.60	−115	−84	10	−85.3	−5.3	−94.4	14.3	−114.0	5.3
0.075	3.25	6.47	9.47	−199	−43	−5	−44.1	14.3	−135.1	76.8	−197.6	−14.3
0.125	3.23	13.57	21.51	−237	−17	−6	−19.4	21.2	−148.2	107.6	−234.7	−21.2
0.175	1.77	22.07	36.94	−254	−2	−5	−4.1	22.1	−150.2	124.1	−252.2	−22.1
0.225	−0.84	31.39	54.84	−266	5	−4	4.0	20.0	−150.0	134.1	−264.2	−20.0
0.275	−4.39	41.12	74.59	−277	9	−3	8.0	16.8	−151.0	142.3	−276.5	−16.8
0.325	−8.72	51.03	95.73	−293	8	−3	7.7	13.3	−155.7	150.1	−292.5	−13.3
0.375	−13.73	60.92	117.88	−311	9	−2	8.3	10.4	−161.6	159.5	−310.6	−10.4
0.425	−19.30	70.66	140.72	−322	11	−1	11.1	7.4	−162.6	166.2	−321.3	−7.4
0.475	−25.38	80.15	163.91	−342	10	−1	10.4	5.2	−170.7	175.9	−341.5	−5.2
0.525	−31.87	89.26	187.16	−353	12	0	12.0	3.1	−173.5	182.3	−352.6	−3.1
0.575	−38.72	97.92	210.15	−367	12	0	12.2	1.5	−178.8	189.6	−366.9	−1.5
0.625	−45.85	106.04	232.62	−383	12	0	11.8	0.1	−185.7	197.4	−383.0	−0.1
0.675	−53.18	113.55	254.32	−388	17	0	16.6	−1.3	−184.3	202.2	−387.8	1.3
0.725	−60.65	120.40	275.04	−401	17	0	16.8	−2.5	−189.6	208.9	−401.1	2.5
0.775	−68.18	126.55	294.64	−398	24	1	23.9	−3.9	−183.4	211.1	−398.3	3.9
0.825	−75.69	132.00	312.99	−396	28	1	27.4	−5.3	−178.9	211.6	−395.8	5.3
0.875	−83.13	136.76	330.04	−391	35	1	35.0	−6.9	−170.8	212.8	−390.5	6.9
0.925	−90.44	140.87	345.79	−383	51	1	51.1	−8.5	−157.4	217.0	−382.9	8.5
0.975	−97.55	144.35	360.24	−367	74	1	73.9	−10.1	−136.1	220.1	−366.3	10.1

**Table 5:** Hole drilling data at the crown (location 1) of the used link.

Depth	ɛ_1_	ɛ_2_	ɛ_3_	*σ_min_*	*σ_max_*	*β*	*σ* on 1	*τ* on 1	*σ* on 2	*τ* on 2	*σ* on 3	*τ* on 3
[mm]	[*μ*ɛ]	[*μ*ɛ]	[*μ*ɛ]	[*MPa*]	[*MPa*]	[°]	[*MPa*]	[*MPa*]	[*MPa*]	[*MPa*]	[*MPa*]	[*MPa*]
0.025	0.28	−1.30	−2.15	−37	12	−11	10.2	9.1	−21.8	22.9	−35.7	−9.1
0.075	−1.77	3.11	5.40	−162	15	−9	10.1	28.6	−102.0	83.4	−156.7	−28.6
0.125	−4.42	8.56	14.93	−213	23	−9	17.4	35.5	−130.6	112.5	−207.6	−35.5
0.175	−8.20	15.67	27.78	−235	31	−8	26.0	37.4	−139.1	127.7	−229.5	−37.4
0.225	−12.99	23.78	42.97	−246	38	−8	33.1	37.6	−141.5	137.0	−241.0	−37.6
0.275	−18.66	32.51	59.84	−256	44	−7	38.9	37.6	−143.5	144.8	−250.7	−37.6
0.325	−25.08	41.62	77.94	−267	47	−7	41.9	37.9	−148.1	152.2	−262.4	−37.9
0.375	−32.13	50.93	96.91	−282	49	−7	44.5	38.7	−154.9	160.8	−277.0	−38.7
0.425	−39.69	60.30	116.42	−288	53	−7	48.8	38.7	−156.3	166.4	−283.9	−38.7
0.475	−47.64	69.58	136.18	−303	55	−6	50.9	39.4	−163.2	174.7	−298.4	−39.4
0.525	−55.87	78.63	155.91	−309	58	−6	53.6	39.0	−164.8	179.3	−305.1	−39.0
0.575	−64.29	87.35	175.36	−318	60	−6	55.9	38.7	−167.7	184.9	−313.8	−38.7
0.625	−72.79	95.61	194.28	−329	61	−6	57.1	38.4	−172.2	190.9	−324.8	−38.4
0.675	−81.30	103.35	212.51	−331	65	−5	61.1	37.3	−170.4	194.2	−327.3	−37.3
0.725	−89.74	110.49	229.88	−340	67	−5	63.2	36.7	−173.3	199.8	−336.4	−36.7
0.775	−98.05	117.02	246.29	−338	71	−5	68.1	35.2	−168.5	201.3	−334.6	−35.2
0.825	−106.18	122.93	261.69	−336	74	−5	70.8	33.8	−164.8	201.8	−332.8	−33.8
0.875	−114.09	128.24	276.04	−332	79	−5	76.8	32.7	−159.2	203.3	−329.8	−32.7
0.925	−121.73	132.97	289.33	−330	91	−4	88.8	32.6	−151.8	208.1	−327.3	−32.6
0.975	−129.08	137.16	301.60	−319	110	−4	107.5	32.6	−136.9	211.8	−316.1	−32.6

**Table 6:** Hole drilling data at the straight part, welded side, (location 2) of the unused link.

Depth	ɛ_1_	ɛ_2_	ɛ_3_	*σ_min_*	*σ_max_*	*β*	*σ* on 1	*τ* on 1	*σ* on 2	*τ* on 2	*σ* on 3	*τ* on 3
[mm]	[*μ*ɛ]	[*μ*ɛ]	[*μ*ɛ]	[*MPa*]	[*MPa*]	[°]	[*MPa*]	[*MPa*]	[*MPa*]	[*MPa*]	[*MPa*]	[*MPa*]
0.025	3.66	2.78	0.71	−280	−220	−15	−223.6	14.9	−264.8	26.3	−276.2	−14.9
0.075	11.65	17.03	20.35	−406	−215	−2	−214.7	5.1	−315.3	95.5	−405.7	−5.1
0.125	19.50	32.79	43.32	−466	−196	0	−196.4	2.0	−333.2	134.9	−466.1	−2.0
0.175	26.83	51.06	71.97	−491	−175	0	−175.2	1.5	−334.7	158.0	−491.1	−1.5
0.225	33.33	70.87	104.55	−502	−158	0	−158.3	1.8	−331.9	171.8	−501.9	−1.8
0.275	38.79	91.42	139.64	−508	−146	0	−145.6	2.0	−328.8	181.3	−508.1	−2.0
0.325	43.15	112.11	176.13	−517	−141	0	−141.1	1.7	−331.0	188.2	−517.5	−1.7
0.375	46.39	132.45	213.14	−526	−136	0	−135.6	0.5	−331.4	195.2	−526.1	−0.5
0.425	48.53	152.07	250.00	−518	−122	0	−122.0	−1.2	−318.9	198.1	−518.1	1.2
0.475	49.66	170.72	286.18	−529	−121	0	−121.3	−3.4	−321.9	204.0	−529.4	3.4
0.525	49.85	188.22	321.28	−528	−115	1	−115.1	−6.1	−315.3	206.3	−527.6	6.1
0.575	49.19	204.44	355.02	−536	−115	1	−115.6	−9.0	−316.6	209.9	−535.4	9.0
0.625	47.80	219.34	387.17	−552	−121	2	−121.8	−12.0	−324.9	215.0	−551.9	12.0
0.675	45.75	232.90	417.59	−552	−116	2	−116.3	−15.0	−318.9	217.5	−551.3	15.0
0.725	43.15	245.14	446.18	−574	−126	2	−126.6	−17.7	−332.1	223.3	−573.1	17.7
0.775	40.07	256.10	472.89	−572	−121	3	−121.8	−20.2	−326.3	224.7	−571.2	20.2
0.825	36.61	265.85	497.69	−580	−127	3	−128.4	−21.9	−331.7	225.3	−578.9	21.9
0.875	32.83	274.45	520.59	−582	−126	3	−126.8	−23.3	−330.3	226.8	−580.4	23.3
0.925	28.80	281.98	541.63	−576	−109	3	−110.1	−24.4	−317.9	232.2	−574.5	24.4
0.975	24.58	288.53	560.85	−551	−76	3	−77.4	−25.0	−288.5	236.1	−549.6	25.0

**Table 7:** Hole drilling data at the straight part, welded side, (location 2) of the used link.

Depth	ɛ_1_	ɛ_2_	ɛ_3_	*σ_min_*	*σ_max_*	*β*	*σ* on 1	*τ* on 1	*σ* on 2	*τ* on 2	*σ* on 3	*τ* on 3
[mm]	[*μ*ɛ]	[*μ*ɛ]	[*μ*ɛ]	[*MPa*]	[*MPa*]	[°]	[*MPa*]	[*MPa*]	[*MPa*]	[*MPa*]	[*MPa*]	[*MPa*]
0.025	1.41	0.77	0.05	−173	−109	−1	−108.8	0.8	−141.6	31.9	−172.6	−0.8
0.075	5.50	9.80	13.99	−279	−124	0	−124.4	0.1	−201.9	77.3	−279.1	−0.1
0.125	9.79	19.80	30.50	−333	−129	4	−129.5	−13.0	−218.1	101.5	−332.6	13.0
0.175	14.33	31.29	51.48	−362	−127	6	−129.7	−25.7	−218.8	114.8	−359.3	25.7
0.225	18.92	43.54	75.66	−380	−126	8	−130.3	−34.2	−218.6	122.5	−375.3	34.2
0.275	23.39	56.20	102.07	−394	−126	8	−132.0	−38.6	−221.6	128.2	−388.3	38.6
0.325	27.60	69.07	129.89	−410	−133	8	−138.4	−39.7	−231.6	132.9	−404.3	39.7
0.375	31.45	82.00	158.53	−426	−138	8	−143.9	−39.2	−243.3	138.6	−421.0	39.2
0.425	34.85	94.84	187.47	−430	−137	7	−141.7	−37.7	−245.9	141.9	−425.5	37.7
0.475	37.74	107.44	216.31	−450	−145	7	−149.2	−37.0	−260.4	148.2	−445.6	37.0
0.525	40.10	119.63	244.73	−460	−147	7	−151.1	−37.0	−266.1	152.0	−455.1	37.0
0.575	41.90	131.26	272.46	−477	−153	7	−157.7	−38.0	−277.1	157.4	−472.5	38.0
0.625	43.13	142.19	299.29	−502	−164	7	−169.3	−40.3	−293.0	164.0	−497.4	40.3
0.675	43.82	152.33	325.04	−515	−167	7	−172.0	−42.8	−298.0	168.8	−509.6	42.8
0.725	43.98	161.63	349.57	−546	−182	7	−187.5	−46.0	−317.6	176.1	−539.6	46.0
0.775	43.63	170.06	372.78	−556	−184	7	−189.8	−47.9	−321.8	179.9	−549.6	47.9
0.825	42.81	177.63	394.58	−571	−192	7	−198.8	−48.7	−333.0	183.0	−564.7	48.7
0.875	41.57	184.41	414.93	−582	−196	7	−202.6	−48.5	−340.7	186.6	−575.8	48.5
0.925	39.94	190.44	433.79	−587	−190	7	−195.8	−47.7	−341.0	192.9	−581.6	47.7
0.975	37.97	195.78	451.17	−574	−168	6	−173.1	−45.3	−325.9	198.1	−569.4	45.3

**Table 8:** Hole drilling data at the straight part, non-welded side, (location 3) of the unused link.

Depth	`ɛ_1_	ɛ_2_	ɛ_3_	*σ_min_*	*σ_max_*	*β*	*σ* on 1	*τ* on 1	*σ* on 2	*τ* on 2	*σ* on 3	*τ* on 3
[mm]	[*μ*ɛ]	[*μ*ɛ]	[*μ*ɛ]	[*MPa*]	[*MPa*]	[°]	[*MPa*]	[*MPa*]	[*MPa*]	[*MPa*]	[*MPa*]	[*MPa*]
0.025	−1.01	−0.50	−1.00	−253	−157	−1	−157.4	2.1	−207.3	47.8	−253.0	−2.1
0.075	10.78	15.74	21.27	−483	−318	5	−318.8	−13.9	−386.6	81.7	−482.2	13.9
0.125	23.69	34.81	47.34	−555	−334	0	−333.9	0.9	−445.5	110.7	−555.3	−0.9
0.175	37.50	58.39	79.49	−564	−293	−5	−294.8	21.3	−450.0	134.0	−562.7	−21.3
0.225	50.25	84.46	115.18	−554	−241	−7	−245.9	37.3	−435.1	151.9	−549.7	−37.3
0.275	61.07	111.54	152.75	−541	−196	−8	−202.5	46.1	−414.8	166.2	−535.0	−46.1
0.325	69.74	138.51	191.10	−536	−169	−8	−175.0	47.7	−400.2	177.5	−530.0	−47.7
0.375	76.37	164.59	229.50	−537	−154	−6	−158.6	43.0	−388.6	187.0	−532.5	−43.0
0.425	81.23	189.28	267.41	−521	−131	−5	−134.4	34.3	−360.6	192.0	−518.4	−34.3
0.475	84.58	212.25	304.43	−530	−130	−3	−131.0	23.6	−353.6	199.0	−529.0	−23.6
0.525	86.65	233.32	340.24	−526	−122	−2	−122.4	13.0	−337.2	201.8	−526.0	−13.0
0.575	87.61	252.42	374.54	−533	−121	0	−121.4	2.8	−329.8	205.6	−532.6	−2.8
0.625	87.57	269.59	407.12	−546	−124	1	−124.5	−4.6	−330.7	210.8	−546.1	4.6
0.675	86.58	284.87	437.76	−536	−108	1	−108.4	−9.6	−312.4	213.6	−535.6	9.6
0.725	84.67	298.38	466.32	−542	−102	1	−102.5	−11.3	−311.0	219.9	−542.2	11.3
0.775	81.90	310.24	492.72	−520	−75	1	−75.4	−10.7	−286.7	222.0	−519.4	10.7
0.825	78.31	320.57	516.94	−503	−55	1	−55.1	−7.7	−271.2	223.8	−502.7	7.7
0.875	74.00	329.50	538.99	−475	−22	1	−21.7	−4.0	−244.5	226.7	−475.2	4.0
0.925	69.10	337.17	558.96	−440	27	0	26.5	−0.4	−206.3	233.2	−439.8	0.4
0.975	63.76	343.70	576.97	−387	89	0	89.4	1.5	−150.2	238.1	−386.8	−1.5

**Table 9:** Hole drilling data at the straight part, non-welded side, (location 3) of the used link.

Depth	ɛ_1_	ɛ_2_	ɛ_3_	*σ_min_*	*σ_max_*	*β*	*σ* on 1	*τ* on 1	*σ* on 2	*τ* on 2	*σ* on 3	*τ* on 3
[mm]	[*μ*ɛ]	[*μ*ɛ]	[*μ*ɛ]	[*MPa*]	[*MPa*]	[°]	[*MPa*]	[*MPa*]	[*MPa*]	[*MPa*]	[*MPa*]	[*MPa*]
0.025	−0.52	−2.79	−2.60	−40	−9	88	−40.4	−1.2	−23.6	−15.6	−9.2	1.2
0.075	3.88	4.39	2.70	−170	−84	−38	−116.8	41.7	−169.0	10.5	−137.7	−41.7
0.125	9.30	14.19	10.59	−268	−125	−30	−161.6	62.2	−259.1	35.3	−232.2	−62.2
0.175	16.44	28.75	23.47	−338	−154	−26	−188.7	71.8	−318.0	57.5	−303.6	−71.8
0.225	24.73	47.07	40.78	−392	−175	−22	−206.6	76.3	−359.8	76.8	−360.2	−76.3
0.275	33.63	68.23	61.82	−436	−191	−20	−220.0	78.7	−392.4	93.7	−407.5	−78.7
0.325	42.72	91.39	85.81	−478	−208	−18	−233.9	80.2	−422.8	108.8	−451.4	−80.2
0.375	51.64	115.79	111.95	−516	−221	−17	−245.7	82.1	−450.3	122.5	−490.8	−82.1
0.425	60.09	140.79	139.51	−534	−221	−16	−244.4	81.9	−459.8	133.4	−511.2	−81.9
0.475	67.86	165.79	167.81	−564	−231	−15	−252.9	82.8	−480.5	144.7	−542.4	−82.8
0.525	74.80	190.32	196.26	−578	−231	−14	−251.1	81.6	−485.8	153.1	−557.2	−81.6
0.575	80.81	213.98	224.38	−594	−233	−13	−252.2	80.2	−493.7	161.3	−574.8	−80.2
0.625	85.84	236.45	251.77	−616	−242	−12	−259.5	78.6	−507.6	169.6	−598.6	−78.6
0.675	89.89	257.51	278.10	−622	−240	−12	−255.8	75.0	−506.5	175.7	−607.2	−75.0
0.725	92.98	276.99	303.16	−645	−252	−11	−265.1	71.8	−520.0	183.1	−631.3	−71.8
0.775	95.17	294.80	326.76	−645	−248	−10	−259.0	66.4	−512.4	187.0	−633.1	−66.4
0.825	96.53	310.91	348.80	−646	−248	−9	−257.8	60.1	−507.1	189.2	−636.2	−60.1
0.875	97.14	325.31	369.21	−643	−244	−8	−251.2	53.9	−497.2	192.1	−635.3	−53.9
0.925	97.09	338.08	387.97	−635	−229	−7	−235.1	49.3	−481.5	197.0	−629.1	−49.3
0.975	96.47	349.28	405.09	−605	−195	−6	−199.5	44.5	−444.4	200.4	−600.4	−44.5

## 2 Experimental Design, Materials, and Methods

The material used in this experiment was from the mooring chain steel grade R4. Two chain links, one referred to as the used and the other as the unused, with the same size (nominal diameter of 114 mm) and made by the same manufacturer where selected. The used link had been exposed to the sea loads for 18 years before the experiment and had some corrosion evidence at the surface. The unused link was 10 years old at the time of the experiment but had never been exposed to any cyclic service loads, as it was laid on the vessel's deck during its service life. The unused link had a relatively smooth surface. The chosen links are pictured in Fig. 1 in [1]. The monotonic mechanical material properties of the tested chain material are provided in [2].

Five locations on each chain link were specified for RS measurements; one in the middle of the bent part (known as the chain crown) and the others at the straight parts to compare RS in the material in the welded side with those in the non-welded side (base material). The locations correspond to the critical locations from a fatigue point of view. Further, marking the measuring locations on the links was rather convenient enabling a point-to-point comparison between the two links and FE simulations. The measurement paths are shown in Fig. 2 in [1]. A local cylindrical coordinate system at every location is defined such that radial stresses are along the R-axis, hoop stresses along the *θ*-axis, and longitudinal stresses along the Z-axis. The strain rosettes for the HD technique are glued such that the axes passing through the strain gauges no. 1 and 3, shown in Fig. 3, are aligned with the hoop and longitudinal directions respectively.

### 2.1 Residual stress measurement techniques

#### 2.1.1 Neutron diffraction

RS can be measured non-destructively via ND that essentially uses the lattice planes in polycrystalline materials as atomic strain gauges. The lattice strain is determined from the shift in the lattice parameter when compared to the corresponding unstressed state. The corresponding stress can then be derived from the deduced lattice strain using the material elastic stiffness. Neutrons offer significantly larger penetration depth (up to several centimeters) in most metallic materials as compared to other diffraction methods and therefore is ideal to probe stresses non-destructively deep within bulk engineering components. Assuming that the measurement axes are aligned with the principal axes, using the measured strains (ɛ) and Hooke's law, one can calculate the RS as follows:

(1) $$\begin{array}{ccc} \varepsilon_{i} & = & \left(d_{i}-d_{0}\right)/d_{0},\;i=r,\theta ,\;Z\;\\ \sigma_{i} & = & \frac{E}{1+\nu }\left[\varepsilon_{i}+\frac{E}{1-2\nu }\left(\varepsilon_{r}+\varepsilon_{\theta }+\varepsilon_{z}\right)\right] \end{array}$$

where *d_i_* are the lattice spacings of the stressed material along the three perpendicular measurement axes and *d*_0_ denotes the average value of the lattice spacing of the stress-free crystalline material. *E* denotes the material's Young's modulus and *ν* is the Poisson's ratio provided in [2].

The uncertainties in the stresses derived (Δ*σ_i_*) is calculating using [3],

(2) $$\begin{array}{c} {\left(\Delta \sigma_{i}\right)}^{2}\;=\;{\left(\frac{A_{err}}{d_{0}}\right)}^{2}\left[\begin{array}{c} B_{err}{\left(\;\Delta d_{i}\;\right)}^{2}\;+\;C_{err}^{2}{\left(\;\Delta d_{0}\;\right)}^{2}\;+\\ \;D_{err}^{2}\left({\left(\;\Delta d_{r}\;\right)}^{2}\;+\;{\left(\;\Delta d_{\theta }\;\right)}^{2}\;+\;{\left(\;\Delta d_{z\;}\right)}^{2}\right) \end{array}\right]\\ A_{err}\;=\;\frac{E}{1+\;\nu },\;B_{err}\;=\;\frac{1}{1\;-\;2\nu },\;C_{err}\;=\;\frac{1\;+\;\nu }{1\;-\;2\nu },\;D_{err}\;=\;\frac{\nu }{1\;-\;2\nu } \end{array}$$

In the first part of the experiment, RS at a few millimeters under the surface at the specified locations on the links were measured using this technique. The work was carried out using ENGIN-X, [4], the time-of-flight neutron diffractometer at ISIS Neutron Source, Rutherford Appleton Laboratory. The diffractometer uses a pulsed polychromatic neutron beam and is optimized for strain measurements with two detectors aligned at fixed scattering angles of 2θ = ±90° that allows simultaneous measurement of two principal strain axes. To obtain 3D RS, the heavy chain links must be positioned in two orientations because in each orientation the lattice spacing along only two perpendicular axes could be measured. Wooden frames were designed for the links to be mounted on the rotation and translation sample stage in ENGIN-X. The experimental setup is shown in Fig. 3 in [1]. A 4 × 4 × 4mm gauge volume was used to enable measurements to be completed at considerable depths below the surface of the links at a reasonably short time considering the limited beamtime access and neutrons’ maximum penetration capability into steels. The center of the gauge volume was positioned at 3 and 4 mm below the surface using theodolites. At such depths, the gauge volume is completely filled with the chain link material and pseudo-strain effects were avoided [5]. Small T-shape samples, as illustrated in Fig. 1, were cut from the straight parts (both the welded side and non-welded side) of the adjacent chain link to the unused chain link and used to determine the lattice spacing of the stress-free material (*d*_0_). The above-mentioned setup provided the RS at 3 and 4mm below the surface, see e.g. Fig. 2.

Due to the beamtime access limitation, the calculation of the RS in locations 2 and 3 is based on a 2-axis measurement (hoop and longitudinal), and thus, the radial strain was set to zero when calculating RS using Eq. (1).

#### 2.1.2 Hole drilling

In this technique, after preparing (dust removal and polishing) the specimen surface, a strain gauge is glued to the specimen and a small hole is drilled (or milled) incrementally into the material at the center of the strain rosette. The released strains at each increment are measured by the strain gauges. The RS are then calculated utilizing the integral method [6], measured released strains, and Hooke's law. Assuming a non-uniform stress profile through the hole in thick workpieces as:

(3) $$\begin{array}{ccc} p_{j} & = & {\left(\varepsilon_{3}+\varepsilon_{1}\right)}_{j}/2\;\;\bar{a}\;P=\frac{E}{1+\nu }p\;{\left(\sigma_{1}\right)}_{j}=P_{j}-Q_{j}\\ q_{j} & = & {\left(\varepsilon_{3}-\varepsilon_{1}\right)}_{j}/2\;\;\bar{b}\;Q=E\;q\;{\left(\sigma_{3}\right)}_{j}=P_{j}+Q_{j}\\ t_{j} & = & {\left(\varepsilon_{3}+\varepsilon_{1}-2\varepsilon_{2}\right)}_{j}/2\;\bar{b}\;T=E\;t\;{\left(\tau_{13}\right)}_{j}=T_{j}\\ & & \;{\left(\sigma_{max}\right)}_{j},{\left(\sigma_{min}\right)}_{j}=P_{j}\pm \sqrt{Q_{j}^{2}+T_{j}^{2}}\\ & & \;\beta_{j}=\frac{1}{2}arctan\left(\frac{T_{j}}{Q_{j}}\right) \end{array}$$

where *j* refers to the serial numbers of the hole depth steps, and ɛ_1_, ɛ_2_, and ɛ_3_ are measured along the three axes of the attached strain gages. ***p,  q***, and ***t*** denote the strain vectors, and **P**, **Q**, and **T** are the incremental transformed stress vectors. $\bar{a}\;$is the calibration constant matrix for isotropic equi-biaxial stress (*P*) and $\bar{b}$ is the calibration constant matrix for 45^∘^ shear stress (*Q*) and *13* shear stress (*T*) and their elements for different hole depths can be extracted from the tables in ASTM E837-13a [7]. *σ_max_* and *σ_min_* are the principal stresses and *β* is the angle measured clockwise from gauge 1 to the maximum principal stress axis, see Fig. 3.

In the second part of the experiment, the RS at and close to the surface of the chain links were measured using this technique at SINTEF in Trondheim. Only RS at the locations around the links (locations 1, 2, and 3 in Fig. 2 in [1]) were measured as the hole drilling instrument couldn't be placed in the limited space between the straight parts of the links. A thin layer of the rust on the surface was removed using very fine Scotch-Brite surface conditioning discs. The strain rosette type 1-RY61-1.5/120K, shown in Fig. 3, connected to an electronic measuring system (Spider8) was used to record the strain variation during incremental hole milling using the MTS-3000 instrument. The rosettes were glued to the surfaces of the links in a way that the axes 1 and 3 of the strain gages were aligned with the hoop (*θ*-axis) and longitudinal (Z-axis) directions respectively, see e.g. Fig. 1 in [1] and Fig. 3. The instrument is equipped with an air turbine enabling the end mill to spin at a speed of 400000 rpm to avoid the introduction of RS during the milling process [8]. The end mills used in the experiment are inverted truncated-cone shaped with flat ends (1-SINTCTT/1), with a maximum shank diameter of 1.60 mm. The above-mentioned setup has provided the measurement of RS at a distance of up to 1mm below the surface. The RS were computed using the measured released strains via EVAL7.14 software.

## Appendix Supplementary materials

Image, application 1
